# Effects of Ellagic Acid on Glucose and Lipid Metabolism: A Systematic Review and Meta-Analysis

**DOI:** 10.1155/2024/5558665

**Published:** 2024-06-17

**Authors:** Xuelian Wang, Xiaotao Zhou, Xinxia Zhang

**Affiliations:** Clinical School of Medicine, Chengdu University of Traditional Chinese Medicine, Chengdu, Sichuan, China

## Abstract

**Background:**

Abnormal glucose and lipid metabolism (GALM) serve as both a cause and an inducer for the development of the disease. Improvement and treatment of GALM are an important stage to prevent the occurrence and development of the disease. However, current clinical treatment for GALM is limited. Ellagic acid (EA), a common polyphenol present in foods, has been shown to improve abnormalities in GALM observed in patients suffering from metabolic diseases.

**Objective:**

This study used a meta-analysis method to systematically assess the effects of EA on GALM.

**Method:**

As of November 8, 2023, a comprehensive search was conducted across 5 databases, namely, PubMed, Embase, Web of Science, Cochrane Library, and Google Scholar to identify randomized controlled trials (RCTs) in which EA served as the primary intervention for diseases related to GALM. The risk of bias within the included studies was assessed according to the Cochrane Handbook. All statistical analyzes were performed using RevMan 5.4 software.

**Results:**

In this study, a total of 482 articles were retrieved, resulting in the inclusion of 10 RCTs in the meta-analysis. The results showed that EA could reduce fasting blood glucose (FBG) (*p* = 0.008), increase insulin secretion (*p* = 0.01), improve insulin resistance index (HOMA-IR) (*p* = 0.003), decrease triglyceride (TG) (*p* = 0.004), and reduce cholesterol (Chol) (*p* = 0.04) and low-density lipoprotein (LDL-c) (*p* = 0.0004). EA had no significant effect on waist circumference (WC), body weight (BW), body mass index (BMI), 2 hours after prandial blood glucose (2 h-PG), total cholesterol (TC), and high-density lipoprotein (HDL-c).

**Conclusions:**

The effect of improvement in glucose and lipids of EA was closely related to the dose and the intervention time. EA can improve GALM caused by diseases. To corroborate the findings of this study and improve the reliability of the results, EA is imperative to refine the research methodology and increase the sample size in future investigations.

## 1. Introduction

Diabetes, a major public health concern throughout the world, affects approximately 500 million people worldwide, with type 2 diabetes mellitus (T2DM) accounting for 90% of these cases [1], there is a significant risk to the physical and mental well-being of individuals. A study shows that nonalcoholic fatty liver disease (NAFLD), a disorder of lipid metabolism, can cause T2DM, particularly in premenopausal women, due to a further slowdown in GALM [2]. Glucose and lipids are essential functional substances in the body, regulated by multiorgan hormonal feedback systems [3]. Impaired metabolism of these substances can lead to metabolic dysfunction. Excessive accumulation of these substances can contribute to obesity, liver disease, hyperlipidemia, T2DM, cardiovascular disease (CVD), and other conditions [4]. Furthermore, the accumulation of glucose and lipids in tumor cells can promote tumor growth and accelerate disease progression [5, 6]. Therefore, maintaining normal glucose and lipid homeostasis is essential for optimal health and wellness.

In recent years, the integration of herbal medicine and nutritionally rich compounds into the management of chronic diseases related to GALM such as T2DM and dyslipidemia have been considered [7–13]. EA, a weakly acidic polyphenol present in fruits and nuts such as blueberries, pomegranates, and walnuts, is easily converted by the body into urolithic acid for absorption [14]. EA has been shown to alleviate inflammation, increase the population of pancreatic beta cells, improve islet function, enhance insulin secretion, and promote serum glucose metabolism [15]. EA reduces chronic inflammatory responses in rats by decreasing serum levels of TNF-*α* and other inflammatory factors [16]. Furthermore, in pancreatic cells, EA mitigates microinflammatory responses by reducing TNF-*α* secretion, increasing cell nuclear antigen levels, and elevating IL-6 concentrations [17]. EA has been found to reduce FBG levels in individuals with T2DM [18]. EA upregulates gene expression of GLUT2, IGF-1, IRS-1, and IRS-2, and EA exerts antiglycation and antioxidant effects by inhibiting lipid peroxidation-mediated malondialdehyde and conjugated dienes [19, 20]. EA acts on *α*-amylase, leading to a decrease in *α*-glucosidase concentration [21], and inhibits glycogen decomposition by reducing glycogen phosphorylase secretion [22]. EA also increases glucose uptake in L-6 cells, stimulates glycogen synthesis, and lowers serum glucose levels [23]. EA can significantly improve the body's glucose metabolism.

EA has been found to promote lipid metabolism by reducing BW and abdominal circumference in obese rats [24]. It has been demonstrated to enhance lipid metabolism by downregulating the expression of the iodothyronine deiodinase 2 and Nr4a1 genes in brown fat, while upregulating the insulin sensitizing gene PPARG coactivator 1 alpha. This regulatory mechanism confers an antioxidative stress effect, inhibits the generation of white fat, and improves lipid metabolism in patients with metabolic syndrome (MetS) [25]. EA accelerates lipid metabolism by reducing the secretion of TG and TC by the liver of mouses [26]. Furthermore, EA can decrease leptin and resistin levels in model rats, increase adiponectin secretion, inhibit adipocyte differentiation, and enhance lipid metabolism [27]. Although the specific mechanism requires further investigation, EA has shown a protective effect against T2DM, CVD, cancer, and neurodegenerative diseases [28, 29]. In addition, it prevents complications of T2DM, such as diabetic retinopathy and diabetic nephropathy [30].

In conclusion, EA has the potential to increase serum GALM in metabolic diseases, thus delay disease progression. However, there remains controversy regarding the clinical use of EA [31]. The lack of clinical data on the natural compound EA and the small sample sizes in the studies are the main issues. To address this issue, a systematic review and meta-analysis of published clinical trial studies was conducted to comprehensively reassess the effects of EA on GALM. This analysis further clarified the relationship between GALM and EA, elucidating the specific contributions of EA and the relative causal effects of associated components.

## 2. Methods

This systematic review and meta-analysis was registered with the International Registry for Prospective Systems Evaluation (PROSPERO, CRD42023404041) according to the PRISMA checklist. This registration was undertaken to ensure transparency and adherence to rigorous research methodology, thereby enhancing the validity and reliability of the study.

### 2.1. Search Strategies

An extensive and meticulous investigation and analysis of scholarly articles was conducted up to December 8, 2023. This investigation spanned several authoritative databases, including PubMed, Cochrane Library, Web of Science, Embase, and Google Scholar. The focal point of this search was the biochemical compound EA and its potential impacts on physiological health indicators. The selection criteria were stringent, focusing on research where key terms such as body weight, body mass index, waist circumference, fasting blood glucose, 2 hours after prandial blood glucose, hemoglobin A1c, insulin resistance index, insulin, and lipid profiles (encompassing total cholesterol, cholesterol, triglycerides, and low- and high-density lipoprotein cholesterol) and were prominently featured in the research titles and abstracts. In addition, the search gives special consideration to studies exploring the levels of leptin and adiponectin, hormones that are crucial in the regulation of metabolic processes.

### 2.2. Inclusion Criteria

(a) The design is a randomised controlled trial (RCT) (cross design or parallel design).
(b) EA or placebo was the primary intervention. The treatment group was treated with EA as the intervention method, while the control group was treated with placebo.
(c) The main outcome index data extracted are waist circumference (WC), body weight (BW), body mass index (BMI), fasting blood glucose (FBG), 2 hours after prandial blood glucose (2 h-PG), insulin, insulin resistance index (HOMA-IR), triglyceride (TG), total cholesterol (TC), cholesterol (Chol), low-density lipoprotein (LDL-c), high-density lipoprotein (HDL-c), leptin, and adiponectin. Outcome index BMI and HOMA-IR calculation formulas are as follows:
  (1) $$\begin{array}{c} \text{BMI }\left(\text{kg}/m^{2}\right)=\frac{\text{Body weight}}{\text{Height}2},\\ \text{HOMA}-\text{IR}=\frac{\text{Fasting blood glucose}*\text{Fasting insulin}}{22.\;5}. \end{array}$$
(d) Involving RCTs published in the English language.

### 2.3. Exclusion Criteria

- Clinical studies of non-EA interventions.
- Publications that lack a diversity of essential data, including thematic information or interventions.
- The results of the study could not be verified or analyzed.
- Duplicate publications.

### 2.4. Data Compilation

#### 2.4.1. Data Screening

The authors utilized EndNote X 9.1 to deduplicate and integrate the retrieved literature. Subsequently, they reviewed the titles and abstracts of the articles to exclude irrelevant literature. Finally, the full text of the remaining articles was downloaded and read to assess their eligibility for inclusion in the analysis. In cases of disagreement between the authors, initial discussions were held to address and resolve the discrepancies. If necessary, a third party would be consulted to assist in resolving the issue. This systematic approach guarantees a meticulous selection of included studies and ensures accurate and reliable analysis.

#### 2.4.2. Data Extraction and Management

The researchers extracted relevant data from all included literature, including basic study information such as the author and date of publication, as well as subject characteristics, including the sample size, gender, age, and country of origin. Furthermore, information on intervention measures was provided, including means, frequency, and duration of treatment, as well as course of treatment.

The primary outcome indicators, corresponding data, the follow-up time, and any reported adverse events were extracted from the studies. Two independent investigators then entered the data into standardized forms (the abovementioned steps are performed by XLW and XTZ). Disagreements were resolved through discussion and, if necessary, consultation with another author. If the information was incomplete or unclear, we contacted the original authors of the studies to obtain the necessary details.

This meticulous process ensures the accuracy, completeness, and reliability of the data collected for analysis, facilitating a comprehensive evaluation of the effects of EA on GALM.

#### 2.4.3. Quality Assessment

Each study's risk of bias was meticulously assessed, referencing the guidelines outlined in the 2014 Cochrane Handbook. This comprehensive evaluation scrutinized seven critical aspects of the study methodology to determine potential biases. These aspects included the generation of a random sequence, ensuring allocation concealment, the blinding process for both participants and personnel, the blinding method applied during outcome assessment, the integrity and completeness of the outcome data reported, the presence of any selective reporting of outcomes, and the identification of any other possible sources of bias.

Based on the previously mentioned criteria, the quality of each study was classified into one of the following three categories: low, high, or unknown risk of bias. This classification is instrumental in assessing the overall quality and dependability of the studies included. Furthermore, it facilitates a more accurate interpretation of the data in our systematic review and meta-analysis.

### 2.5. Data Synthesis and Statistical Analysis

RevMan 5.4 software was used utilized for the meta-analysis. The analysis involved a 95% confidence interval (95% CI) and calculation of the standard mean difference (SMD) for continuous variables. The *I*^2^ statistic was used to assess heterogeneity between studies. In cases where heterogeneity tests showed a *p* value of <0.01 and/or an *I*^2^ value of <50%, random effects models were applied for combined effect estimates. Conversely, fixed-effects models were used for studies exhibiting low heterogeneity, as indicated by a *p* value >0.10 and an *I*^2^ value <50%. To ensure the reliability of the study, we analyzed subgroups of articles with at least 10 experimental data points. We performed the subgroup analysis using RevMan 5.4 software.

Statistically significant was considered achieved when the *p* value was <0.05. In cases where the meta-analysis comprised at least 10 studies, a funnel plot analysis was performed using RevMan 5.4 software to assess publication bias. This assessment helps to evaluate the possible impact of publication bias on the overall results of the meta-analysis.

## 3. Result

### 3.1. Literature Search and Retrieval

Initially, a total of 482 articles were retrieved from major English databases, consisting of 478 articles identified through the literature search and an additional 4 articles from other sources. After the removal of duplicates and an initial screening, 40 studies were found to be replicated and accordingly excluded from further analysis. This exclusion process involved the removal of 384 studies that did not meet the criteria, which included case reports, reviews, meta-analyses, noninterventional basic studies, and nonclinical controlled trials.

**Figure 1:**
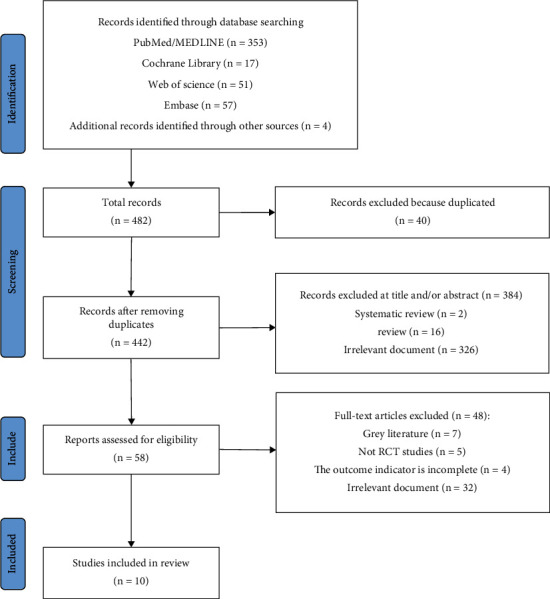
Flowchart of included studies.

In this meta-analysis, 10 RCTs that fulfilled the inclusion criteria were analyzed. They were selected based on their relevance to the research question and compliance with pre-established inclusion criteria. To visually represent the screening process, a flowchart is provided to depict the screening process visually, detailing the number of articles reviewed at each phase and the reasons for their exclusion. The flowchart of the research and screening process is illustrated in Figure 1. Specific search methods and search results are shown in Table 1.

### 3.2. Characteristics of Clinical Studies

The systematic review included a total of 10 studies (involving 419 patients) [32–41]. The median age of the participants ranged from 24 to 63 years. 2 studies focused on MetS [34, 37], 2 studies on CVD [34, 39], and 6 studies on T2DM [32], polycystic ovary syndrome (PCOS) [33], irritable bowel syndrome (IBS) [38], NAFLD [36], obese, overweight [35, 36], and women with high blood pressure after menopause (BP of women after menopause) [40]. In the experimental group, EA was the primary intervention, while the control group received a placebo. The intervention period ranged from 4 to 12 weeks. 6 studies make that made EA into capsules [32, 33, 35, 37–39], and 5 studies were conducted by means of administration with pills [32, 33, 36–38], powered [34], drinks [42], and milkshake [34]. 5 intervention groups were given EA [32, 33, 36–38], 3 are pomegranate fruits [35, 39, 41] 2 are blueberries [34, 40]. The control group received a placebo. Follow-up information was not mentioned in 5 cases. Detailed information on the included RCTs is shown in Table 2.

### 3.3. Risk of Bias

The risk of bias in the RCTs included in the study was evaluated using the bias risk assessment criteria outlined in the Cochrane Manual. The assessment focused on the following two aspects: the generation of randomly assigned sequences (selective bias) and allocation hiding. 7 studies used the random number method, 1 study used the stratified random method, and 2 studies used the block random method. This information is crucial to evaluate the potential for selective bias in included RCTs.

Regarding the concealment of the allocation, five studies reported using sealed containers to hide random allocation schemes, suggesting a lower risk of bias. However, five studies did not provide details on the methods used for concealment of allocation, indicating a higher risk of bias in these studies.

**Figure 2:**
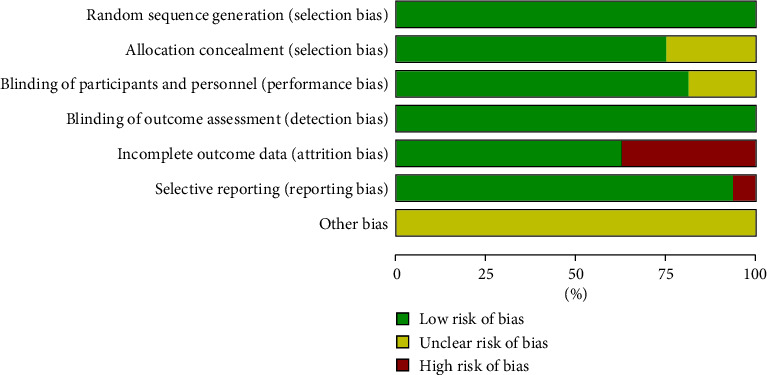
Risk of bias graph.

**Figure 3:**
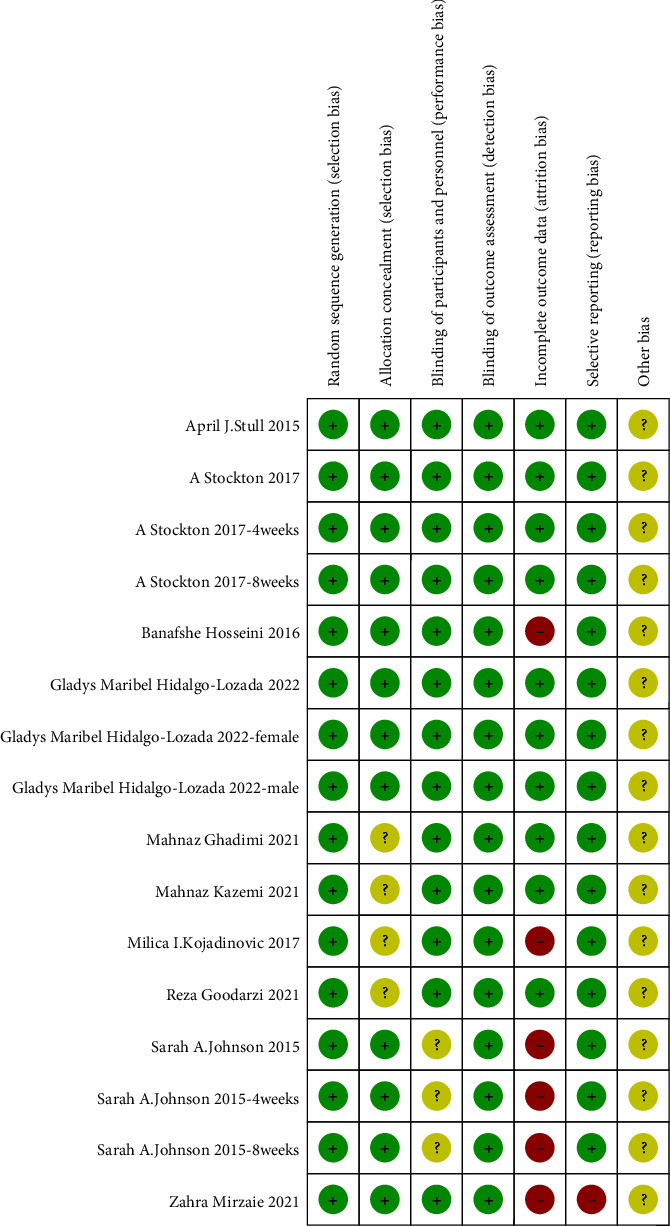
Risk of bias summary.

Figures 2 and 3 graphically represent the results of the risk of bias assessment, showing the level of bias present in the included RCTs. These figures assist the reader in interpreting the overall quality and potential limitations of the studies included in the meta-analysis.

### 3.4. Meta-Analysis

The meta-analysis incorporated 10 RCTs involving a total of 419 patients. Random-effects models were used to analyze the effects of EA on various metabolic indices before and after the intervention. This approach allows for the summarization of the results while taking into account potential heterogeneity among the included studies. This methodology provides a more robust and generalizable estimate of the effects of EA on metabolic indices. To fully appreciate the implications of the study findings, it is necessary to provide the specific results of the meta-analysis, including the effect sizes and their associated confidence intervals. These results could provide information on the potential benefits of the EA intervention for metabolic health and shed light on its overall impact on metabolic indices.

### 3.5. Main Outcome

Due to the different diseases included, we performed a pooled meta-analysis of the characteristics of the baseline population included. The results showed that there were no significant differences in baseline age, BW, BMI, FBG, 2 h-PG, insulin, HOMA-IR, TG, TC, Chol, LDL-c, and HDL-c of the patients included in all studies, as shown in Table 3.

**Figure 4:**
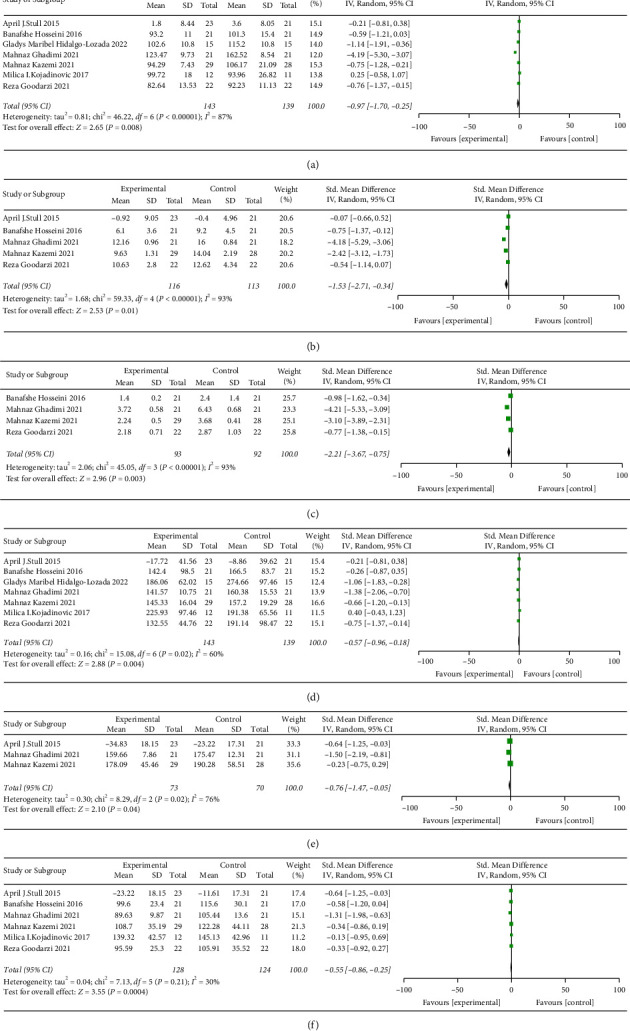
Meta-analysis of EA improving glucose and lipid metabolism. (a) Fasting blood glucose (FBG), (b) insulin, (c) insulin resistance index (HOMA-IR), (d) triglyceride (TG), (e) cholesterol (Chol), and (f) low-density lipoprotein (LDL-c).

The meta-analysis of the effects of EA on GALM following the intervention showed specific results, as shown in Figure 4.

Upon the completion of the data analysis, it was determined that EA significantly decreased FBG (*p*=0.008, SMD: −0.97, 95% Cl: −1.70–0.25, and *I*^2^ = 87%) (Figure 4(a)). EA increased insulin secretion (*p*=0.01, SMD: −1.53, 95% Cl: −2.71–−0.34, and *I*^2^ = 93%) (Figure 4(b)) and lower HOMA-IR (*p*=0.003, SMD: −2.21, 95% Cl: −3.67–−0.75, and *I*^2^ = 93%) (Figure 4(c)). TG was decreased by EA (*p*=0.004, SMD: −0.57, 95% Cl: −0.96–−0.18, and *I*^2^ = 60%) (Figure 4(d)). The EA group significantly reduced Chol (*p*=0.04, SMD: −0.76, 95% Cl: −1.47–−0.05, and *I*^2^ = 76%) (Figure 4(e)). EA significantly reduced LDL-c (*p*=0.0004, SMD: −0.55, 95% Cl: −0.86–−0.25, and *I*^2^ = 30%) (Figure 4(f)). EA had no significant effect on reducing WC (*p*=0.40, SMD: −0.12, 95% Cl: −0.41–0.16, and *I*^2^ = 28%), BW (*p*=0.89, SMD: −0.01, 95% Cl: −0.19–0.17, and *I*^2^ = 0%), BMI (*p*=0.52, SMD: −0.06, 95% Cl: −0.24–0.12, and *I*^2^ = 1%), 2h-PG (*p*=0.51, SMD: −1.65, 95% Cl: −6.53–3.23, and *I*^2^ = 98%), TC (*p*=0.30, SMD: −0.32, 95% Cl: −0.93–0.29, and *I*^2^ = 75%), and HDL-c (*p*=0.13, SMD: 0.32, 95% Cl: −0.09–0.72, and *I*^2^ = 68%). In conclusion, EA did not lead to a reduction in WC, BW, and BMI. EA improved FBG levels, but it did not affect 2h-PG levels. EA can increase insulin secretion, reduce HOMA-IR, and improve insulin resistance in patients. EA can reduce the levels of TG, Chol, and LDL-c, but it has no significant effect on TC and HDL-c. The funnel plots are shown in Supplementary Figure S1.

### 3.6. Subgroup and Sensitivity Analyses

To ensure the reliability of the study, we analyzed data in subgroups based on the following three main aspects: mean age (≥40, <40), dosing time (≥8 weeks, <8 weeks), and dosing dose (≥180 mg/d, <180 mg/d). Subgroup analysis revealed that when EA was the primary intervention; it did not result in a significant reduction in WC and BW among patients. When the intervention lasted more than 8 weeks, EA reduced the BMI of the patients (*p*=0.02, SMD: −0.77, 95% Cl: −1.4 4–−0.10, andd *I*^2^ = 48%) and HDL-c (*p* < 0.00001, SMD: 0.65, 95% Cl: 0.36–0.93, and *I*^2^ = 0%). At the same time, EA can significantly reduce FBG in patients (*p*=0.007, SMD: −1.62, 95% Cl: −2.79–−0.45, and *I*^2^ = 91%), TG (*p* < 0.00001, SMD: −0.91, 95% Cl: −1.23–−0.59, and *I*^2^ = 2%), and TC (*p*=0.02, SMD: −0.77, 95% Cl: −1.44–−0.10, and *I*^2^ = 48%). When the intervention lasted no longer than 8 weeks, EA significantly reduced LDL-c in patients (*p*=0.009, SMD: −0.51, 95% Cl: −0.89–−0.21, and *I*^2^ = 0%). When the intervention dose exceeded 180 mg/d, EA significantly reduced LDL-c in patients (*p*=0.004, SMD: −0.55, 95% Cl: −0.93–−0.17, and *I*^2^ = 44%). Meanwhile, EA significantly reduced LDL-c in patients over 40 years of age (*p*=0.001, SMD: −0.61, 95% Cl: −0.98–−0.24, and *I*^2^ = 37%). The results of the analysis for specific subgroups are shown in Table 4.

By deleting the included literature one by one and conducting a sensitivity analysis, it was shown that EA had a low sensitivity to WC, BW, BMI, and HDL-c and a high sensitivity to FBG, TG, TC, LDL-c, insulin, and HOMA-IR. However, the effect on Chol (*p*=0.18, SMD: −0.84, 95% Cl: −2.09–0.4, and *I*^2^ = 88%) after excluding April 2015 is unstable. The specific analysis results are shown in Supplementary Table S1.

## 4. Discussion

This meta-analysis examined the effect of EA on GALM in MetS. The results of the meta-analysis demonstrated that EA had a remarkable effect on the reduction of FBG, TG, TC, LDL-c, insulin, and HOMA-IR. EA had no significant effect on abdominal circumference, BW BMI, 2 h-PG, TC, and HDL-c. In addition, the results of the sensitivity analysis demonstrated that EA has an unstable effect on cholesterol. In conclusion, EA can improve the abnormal metabolism of GALM in serum by regulating the changes in FBG, insulin, HOMA-IR, TG, and LDL-c.

Human energy intake involves the conversion of carbohydrate into glucose. Lipids, which include fats and other molecules, are crucial components of cell membranes and serve as a primary energy source for the body [41]. Research has shown that altered GALM can cause inflammation, hyperglycemia, and hyperlipidemia, which ultimately results in metabolic disorders such as diabetes and hyperlipidemia [43, 44]. In severe cases, CVD, malignant, tumors, and neuroanatomic diseases can arise [45–48]. Therefore, ensuring proper GALM is extremely important for the prevention and management of diseases.

EA is a polyphenolic compound which is prevalently found in a variety of sources such as fruits grains and nuts [49]. It is known for its high bioavailability in the human body [16]. Recently, there has been a growing body of research focusing on EA. It showed antioxidant and anti-inflammatory properties [50], protects the liver [51], enhances neuronal activity [52], and has anti-T2DM, antiatherosclerosis, and anticancer effects [53]. In addition, it delays the development of chronic diseases. Research on the mechanism of glucose metabolism has shown that EA reduces serum glucose levels in rats by activating 5′AMP-activated protein kinase [54]. It also mitigates glucose-induced damage to pancreatic mesangial cells by activating the PI3K/Akt/FOXO3 signaling pathway, restoring pancreatic cell function, and reducing serum glucose concentration [20]. EA improves insulin resistance in hyperuricemic rats by activating C1q/tumor necrosis factor-associated protein-3 and inhibiting ATP citrate lyase, thereby increasing glucose metabolism [55]. EA reduces serum glucose levels by decreasing advanced glycation end products and increasing glucose utilization [56]. It inhibits the expression of glucose-*α*-glucosidase activity, leading to a decrease in serum glucose concentration [20]. Furthermore, EA acts as an antioxidant by regulating the expression of peroxisome proliferator-activated receptors-*γ* and insulin receptor subunit-1. This improves systemic inflammation in white adipose tissue, increases lipid metabolism, improves insulin sensitivity, and reduces serum glucose concentration. Furthermore, EA can prevent the death of beta cells, increase their quantity, and improve their function by decreasing the expression of the insulin-sensitive gene caspase 3 [57]. This results in elevated insulin secretion and an overall antidiabetic effect. The effectiveness of EA in reducing glucose levels is comparable to that of metformin. Several meta-analyses have indicated that EA contributes to lowering FBG, enhancing pancreatic islet functionality, increasing insulin responsiveness to serum glucose, and boosting the overall metabolism of serum glucose, aligning with the findings of other relevant meta-analytical studies [44, 58].

In general, studies indicate that EA exerts beneficial effects on lipid metabolism [59]. EA increases the uptake of LDL-c and the secretion of the apoA-1 apolipoprotein, while maintaining HDL-c within the normal range [60]. Administration of EA in mice resulted in a reduction in FBG levels and an increase in serum adiponectin [31]. In addition, EA reduces the concentration of TG and TC secreted by the liver, thus enhancing lipid metabolism [24]. This is achieved by enhancing the expression of the peroxisome proliferator-activated receptor-*γ* and CCAAT/enhancer binding protein-*α* protein and promoting EA oxidation [61]. EA decreased TG and Chol levels and improved liver lipid metabolism in aged liver by upregulating the expression of silent information regulator 1, adenosine 5-monophosphate-activated protein kinase, sterol regulatory element binding protein 1, peroxisome proliferator-activated receptors, and phosphorylated AMPK in the liver [62]. The meta-analysis found that EA effectively reduces serum TG, Chol, and LDL-c concentrations and improves lipid metabolism in patients. However, EA was found to be less effective in affecting HDL-c levels. Subgroup analysis suggests that this can be attributed to the different types of disease included in the studies.

The results of the meta-analysis indicate that EA supplementation does not result in a significant change in WC, BW, TC, and HDL-c levels in patients. However, subgroup analysis suggests that EA can reduce BMI, TC, and HDL-c levels in patients after correlation with RCTs but does not lead to significant changes in WC and BW. The degree of improvement in insulin resistance was found to be related to the dose and duration of the EA intervention. In addition, no adverse reactions were reported in these clinical trials. These findings suggest that EA supplementation has a significant impact on improving serum GALM in patients. The evidence indicates that EA has a positive impact on serum GALM.

### 4.1. Limitations

This systematic review and meta-analysis have several limitations that should be acknowledged. One major limitation is the significant heterogeneity observed in most of the pooled outcomes. Subgroup analyzes, stratified by age, dose, and duration of administration, failed to substantially mitigate heterogeneity in pooled results for most of the targeted endpoints. However, it should be noted that the stability of BMI, FBG, TG, TC, HDL-c, insulin, and HOMA-IR results can be influenced by the dose and duration of administration, which requires further investigation.

In addition, the low methodological quality of the included studies may have slightly reduced the reliability of the combined results. The small sample size of articles analyzed in this study makes it challenging to establish definitive conclusions. The literature reviewed was limited to publications in English, which could restrict generalizability and EA supplementation to ethnic groups whose primary language is not English. Based on the findings of this study, well-designed, rigorous RCTs with larger populations are needed to investigate the role of EA and the relationship between dose, duration of the intervention, therapeutic efficacy, and adverse reactions. Furthermore, for the management of patients with elevated serum glucose and lipid levels, a focus on long-term patient care is essential. To date, there have been limited clinical investigations on oral effects of EA in such patients, and future large-scale, well-designed trials will be necessary to confirm the therapeutic mechanism of EA.

## 5. Conclusions

In conclusion, our findings indicate that EA supplementation can effectively lower serum glucose levels and improve lipid metabolism in patients, indicating potential benefits for the management of abnormal GALM. This systematic review sheds light on the role of EA supplementation in GALM. We hope that this study will provide clinicians with additional options to incorporate EA into their therapeutic strategies.

## Figures and Tables

**Table 1:** Search methods of meta-analysis.

Databases	Retrieval criteria	Search results
PubMed	Ellagic acid	353
Web of Science	Ellagic acid	51
Embase	Ellagic acid	57
Cochrane Library	Ellagic acid	17
Other sources	Ellagic acid	4

**Table 2:** Characteristics of included studies.

Authors	Year	Country	Disease	Total		Dosing dose	Dosing time (weeks)	Adverse event	Follow-up
				Intervention	Control				
Hidalgo-Lozada et al.	2022	Mexico	MetS	15	15	500 mg/12 h	12	Feces softening	None
Ghadimi et al.	2021	Iran	T2DM	21	21	180 mg/d	8	0	Mentioned
Kazemi et al.	2021	Iran	PCOS	29	28	200 mg/d	8	0	Mentioned
Mirzaie et al.	2021	Russia	IBS	22	21	180 mg/d	8	0	None
Goodarzi et al.	2021	Iran	NFLD	22	22	180 mg/12 h	12	0	Mentioned
Kojadinovic et al.	2017	Serbia	MetS	12	11	300 ml/d	6	—	None
Stockton et al.	2017	UN	CDV	28	26	1Pomegranate/d	8	—	None
Hosseini et al.	2016	Iran	Obese, overweight	21	21	40 mg/d	4	—	Mentioned
Stull et al.	2015	USA	CDV	23	21	45 g/d	6	—	None
Johnson et al.	2015	USA	BP	20	20	22 g/d	8	—	Mentioned

**Table 3:** Baseline analysis of meta-analysis.

Variables	Number of SD included	Total	SMD	95% CI	*p*	*I* ^2^
Age	8	354	−0.02	[−0.28, 0.23]	0.87	32
WC	5	182	0.33	[0.03, 0.62]	0.03	0
BW	9	399	0.06	[−0.13, 0.26]	0.53	0
BMI	10	422	0.01	[−0.19, 0.21]	0.90	7
FBG	7	284	0.06	[−0.17, 0.29]	0.61	0
2 h-PG	2	72	0.01	[−0.60, 0.62]	0.98	43
Insulin	5	229	0.07	[−0.19, 0.33]	0.61	0
HOMA-IR	3	143	−0.01	[−0.34, 0.32]	0.95	0
TG	7	284	0.12	[−0.11, 0.36]	0.30	0
TC	5	183	−0.15	[−0.44, 0.14]	0.33	0
Chol	3	143	0.13	[−0.20, 0.45]	0.45	0
LDL-c	6	252	0.02	[−0.23, 0.27]	0.86	0
HDL-c	8	316	−0.08	[−0.30, 0.15]	0.50	0

**Table 4:** Subgroup analysis of meta-analysis.

	Variable	Number of included	*N*	SMD	95% CL	*p*	*I* ^2^
WC	*Dosing time*						
	≥8 weeks	6	248	−0.19	[−0.45, 0.08]	0.17	8
	<8 weeks	1	23	0.57	[−0.27, 1.41]	0.18	

BW	*Mean age*						
	≥40	6	239	−0.09	[−0.35, 0.16]	0.49	0
	<40	5	250	0.06	[−0.19, 0.31]	0.63	0
	*Dosing dose*						
	≥180 mg/d	10	447	−0.04	[−0.23, 0.14]	0.66	0
	<180 mg/d	1	42	0.3	[−0.31, 0.91]	0.33	
	*Dosing time*						
	≥8 weeks	9	403	−0.05	[−0.25, 0.14]	0.61	0
	<8 weeks	2	86	0.17	[−0.26, 0.59]	0.44	

BMI	*Mean age*						
	≥40	7	261	−0.07	[−0.37, 0.24]	0.68	36
	<40	4	208	−0.04	[−0.31, 0.23]	0.79	0
	*Dosing dose*						
	≥180 mg/d	10	427	−0.08	[−0.28, 0.12]	0.44	7
	<180 mg/d	1	42	0.13	[−0.47, 0.74]	0.67	
	*Dosing time*						
	≥8 weeks	8	260	−0.77	[−1.44, −0.10]	0.02	48
	<8 weeks	3	109	−0.01	[−0.89, 0.86]	0.97	80

FBG	*Mean age*						
	≥40	5	183	−1.15	[−2.28, −0.01]	0.05	91
	<40	2	99	−0.68	[−1.09, −0.27]	0.001	0
	*Dosing dose*						
	≥180 mg/d	6	240	−1.06	[−1.93, −0.19]	0.02	89
	<180 mg/d	1	42	−0.59	[−1.21, 0.03]	0.06	
	*Dosing time*						
	≥8 weeks	4	173	−1.62	[−2.79, −0.45]	0.007	91
	<8 weeks	3	109	−0.25	[−0.68, 0.19]	0.27	23

TC	*Dosing dose*						
	≥180 mg/d	4	141	−0.19	[−0.95, 0.57]	0.62	79
	<180 mg/d	1	42	−0.79	[−1.42, −0.16]	0.01	
	*Dosing time*						
	≥8 weeks	2	74	−0.77	[−1.44, −0.10]	0.02	48
	<8 weeks	3	109	−0.01	[−0.89, 0.86]	0.97	80

TG	*Mean age*						
	≥40	5	183	−0.61	[−1.19, −0.04]	0.04	71
	<40	2	99	−0.49	[−0.89, −0.08]	0.02	0
	*Dosing dose*						
	≥180 mg/d	6	240	−0.63	[−1.07, −0.18]	0.006	64
	<180 mg/d	1	42	−0.26	[−0.87, 0.35]	0.4	
	*Dosing time*						
	≥8 weeks	4	173	−0.91	[−1.23, −0.59]	<0.00001	2
	<8 weeks	3	109	−0.1	[−0.48, 0.27]	0.59	0

Chol	*Mean age*						
	≥40	2	86	−1.06	[−1.90, −0.21]	0.01	70
	<40	1	57	−0.23	[−0.75, 0.29]	0.39	
	*Dosing time*						
	≥8 weeks	2	99	−0.84	[−2.09, 0.40]	0.18	88
	<8 weeks	1	44	−0.64	[−1.25, −0.03]	0.04	

LDL-c	*Mean age*						
	≥40	5	195	−0.61	[−0.98, −0.24]	0.001	37
	<40	1	57	−0.34	[−0.86, −0.25]	0.21	
	*Dosing dose*						
	≥180 mg/d	5	210	−0.55	[−0.93, −0.17]	0.004	44
	<180 mg/d	1	42	−0.58	[−1.20, 0.04]	0.07	
	*Dosing time*						
	≥8 weeks	3	143	−0.63	[−1.22, −0.04]	0.04	67
	<8 weeks	3	109	−0.51	[−0.89, −0.21]	0.009	0

HDL-c	*Mean age*						
	≥40	6	213	0.35	[−0.23, 0.92]	0.24	76
	<40	2	99	0.21	[−0.19, 0.60]	0.3	0
	*Dosing dose*						
	≥180 mg/d	7	270	0.35	[−0.12, 0.81]	0.14	71
	<180 mg/d	1	42	0.08	[−0.52, 0.69]	0.79	
	*Dosing time*						
	≥8 weeks	5	203	0.65	[0.36, 0.93]	<0.00001	0
	<8 weeks	3	109	−0.3	[−0.98, 0.39]	0.4	67

Insulin	*Mean age*						
	≥40	3	130	−1.53	[−3.44, 0.38]	0.12	95
	<40	2	99	−1.58	[−3.22, 0.06]	0.06	92
	*Dosing dose*						
	≥180 mg/d	4	187	−1.75	[−3.30, −0.19]	0.03	95
	<180 mg/d	1	42	−0.75	[−1.37, −0.12]	0.02	
	*Dosing time*						
	≥8 weeks	3	143	−2.33	[−4.25, −0.41]	0.02	95
	<8 weeks	2	86	−0.4	[−1.06, 0.26]	0.24	58

HOMA-IR	*Mean age*						
	≥40	2	76	−2.45	[−5.82, 0.92]	0.15	96
	<40	2	99	−2.03	[−4.11, 0.05]	0.06	94
	*Dosing dose*						
	≥180 mg/d	3	143	−2.65	[−4.70, −0.61]	0.01	95
	<180 mg/d	1	42	−0.98	[−1.62, −0.34]	0.003	
	*Dosing time*						
	≥8 weeks	3	143	−2.65	[−4.70, −0.61]	0.01	95
	<8 weeks	1	42	−0.98	[−1.62, −0.34]	0.003	

## Data Availability

No data were used to support the findings of this study.

## Supplementary Materials

Supplementary Materials Supplementary Table S1: sensitivity analysis of meta-analysis. Supplementary Figure S1: meta-analysis of EA improving glucose and lipid metabolism.
