# Supplementary material for: Effects of Ellagic Acid on Glucose and Lipid Metabolism: A Systematic Review and Meta-Analysis
Source: J Nutr Metab. 2024 Jun 17;2024:5558665. doi: 10.1155/2024/5558665 (PMC11196188; doi:10.1155/2024/5558665)
Supplement: Supplementary Materials — Supplementary Table S1: sensitivity analysis of meta-analysis. Supplementary Figure S1: meta-analysis of EA improving glucose and lipid metabolism. [file 5558665.f1.zip › Table S1. Sensitivity analysis of meta-analysis..pdf]

Sensitivity analysis of meta-analysis.

Indicators	Excluding study	SMD	95%CL	P	I^2
WC	A-4weeks 2017	-0.15	[-0.50,0.20]	0.4	38
	A-8weeks 2017	-0.12	[-0.47,0.23]	0.51	40
	Gladys-female 2022	-0.13	[-0.46,0.21]	0.45	40
	Gladys-male 2022	-0.03	[-0.28,0.22]	0.81	0
	Milica 2017	-0.19	[-0.45,0.08]	0.17	8
	Sarah-4weeks 2015	-0.08	[-0.41,0.25]	0.63	34
	Sarah-8weeks 2015	-0.16	[-0.49,0.16]	0.33	34
BW	April 2015	-0.02	[-0.20,0.17]	0.85	0
	A-4weeks 2017	-0.02	[-0.21,0.17]	0.82	0
	A-8weeks 2017	0.02	[-0.17,0.21]	0.85	0
	Banafshe 2016	-0.04	[-0.23,0.14]	0.66	0
	Gladys 2022	-0.01	[-0.20,0.17]	0.88	0
	Mahnaz G2021	0	[-0.19,0.19]	0.99	0
	Mahnaz K2021	-0.04	[-0.23,0.15]	0.7	0
	Reza 2021	-0.04	[-0.22,0.15]	0.69	0
	Sarah-4weeks 2015	0.02	[-0.16,0.21]	0.81	0
	Sarah-8weeks 2015	0.01	[-0.17,0.20]	0.88	0
	Zahra 2021	-0.02	[-0.21,0.17]	0.83	0
BMI	April 2015	-0.07	[-0.27,0.13]	0.5	10
	A-4weeks 2017	-0.06	[-0.27,0.14]	0.55	11

	A-8weeks 2017	-0.06	[-0.27,0.14]	0.55	11
	Banafshe 2016	-0.08	[-0.28,0.12]	0.44	7
	Gladys 2022	-0.04	[-0.22,0.15]	0.72	0
	Mahnaz G 2021	-0.04	[-0.25,0.16]	0.67	9
	Mahnaz K2021	-0.04	[-0.24,0.16]	0.7	8
	Milica 2017	-0.11	[-0.30,0.08]	0.25	0
	Sarah-4weeks 2015	-0.03	[-0.22,0.16]	0.76	0
	Sarah-8weeks 2015	-0.04	[-0.23,0.16]	0.71	6
	Zahra 2021	-0.08	[-0.28,0.12]	0.43	7
FBG	April 2015	-0.12	[-1.96,-0.28]	0.009	88
	Banafshe 2016	-1.06	[-1.93,-0.19]	0.02	89
	Gladys 2022	-0.96	[-1.79,-0.13]	0.02	89
	Mahnaz G 2021	-0.55	[-0.89,-0.22]	0.001	37
	Mahnaz K 2021	-1.04	[-1.93,-0.14]	0.02	89
	Milica 2017	-1.17	[-1.94,-0.39]	0.003	87
	Reza 2021	-1.03	[-1.90,-0.16]	0.02	89
TG	April 2015	-0.64	[-1.08,-0.19]	0.005	63
	Banafshe 2016	-0.63	[-1.07,-0.18]	0.006	64
	Gladys 2022	-0.5	[-0.93,-0.08]	0.02	63
	Mahnaz G 2021	-0.45	[-0.80,-0.10]	0.01	44
	Mahnaz K 2021	-0.55	[-1.03,-0.08]	0.02	67

	Milica 2017	-0.69	[-1.04,-0.34]	0.0001	46
	Reza 2021	-0.54	[-1.00,-0.08]	0.02	66
TC	April 2015	-0.39	[-1.16,0.39]	0.33	79
	Banafshe 2016	-0.19	[-0.95,0.57]	0.62	79
	Gladys 2022	-0.29	[-1.07,0.49]	0.47	81
	Milica 2017	-0.58	[-1.04,-0.12]	0.01	52
	Reza 2021	-0.12	[-0.76,0.52]	0.71	71
Chol	April 2015	-0.84	[-2.09,0.40]	0.18	88
	Mahnaz G 2021	-0.76	[-1.47,-0.05]	0.04	76
	Mahnaz K 2021	-1.06	[-1.90,-0.21]	0.01	70
LDL-c	April 2015	-0.54	[-0.91,-0.16]	0.005	43
	Banafshe 2016	-0.55	[-0.93,-0.17]	0.004	44
	Mahnaz G 2021	-0.42	[-0.70,-0.15]	0.003	0
	Mahnaz K 2021	-0.61	[-0.98,-0.24]	0.001	37
	Milica 2017	-0.61	[-0.94,-0.28]	0.0003	34
	Reza 2021	-0.6	[-0.97,-0.24]	0.001	38
HDL-c	April 2015	0.36	[-0.10,0.82]	0.12	70
	Banafshe 2016	0.35	[-0.12,0.81]	0.14	71
	Gladys 2022-female	0.29	[-0.17,0.75]	0.22	72
	Gladys 2022-male	0.27	[-0.18,0.73]	0.24	72
	Mahnaz G 2021	0.21	[-0.20,0.63]	0.31	64
	Mahnaz K 2021	0.31	[-0.17,0.80]	0.21	72

	Milica 2017	0.47	[0.17,0.77]	0.002	36
	Reza 2021	0.23	[-0.21,0.66]	0.3	67
Insulin	April 2015	-1.91	[-3.29,-0.53]	0.007	93
	Banafshe 2016	-1.75	[-3.30,-0.19]	0.03	95
	Mahnaz G 2021	-0.93	[-1.88,0.02]	0.05	89
	Mahnaz K 2021	-1.59	[-2.58,-0.01]	0.05	93
	Reza 2021	-1.80	[-3.33,-0.26]	0.05	95
HOMA-IR	Banafshe 2016	-2.65	[-4.70,-0.61]	0.01	95
	Mahnaz G 2021	-1.6	[-2.94,-0.25]	0.02	92
	Mahnaz K 2021	-1.91	[-3.59,-0.24]	0.03	93
	Reza 2021	-2.72	[-4.61,-0.84]	0.005	94
