# Supplementary figures and images for: Effects of Ellagic Acid on Glucose and Lipid Metabolism: A Systematic Review and Meta-Analysis
Source: J Nutr Metab. 2024 Jun 17;2024:5558665. doi: 10.1155/2024/5558665 (PMC11196188; doi:10.1155/2024/5558665)

A

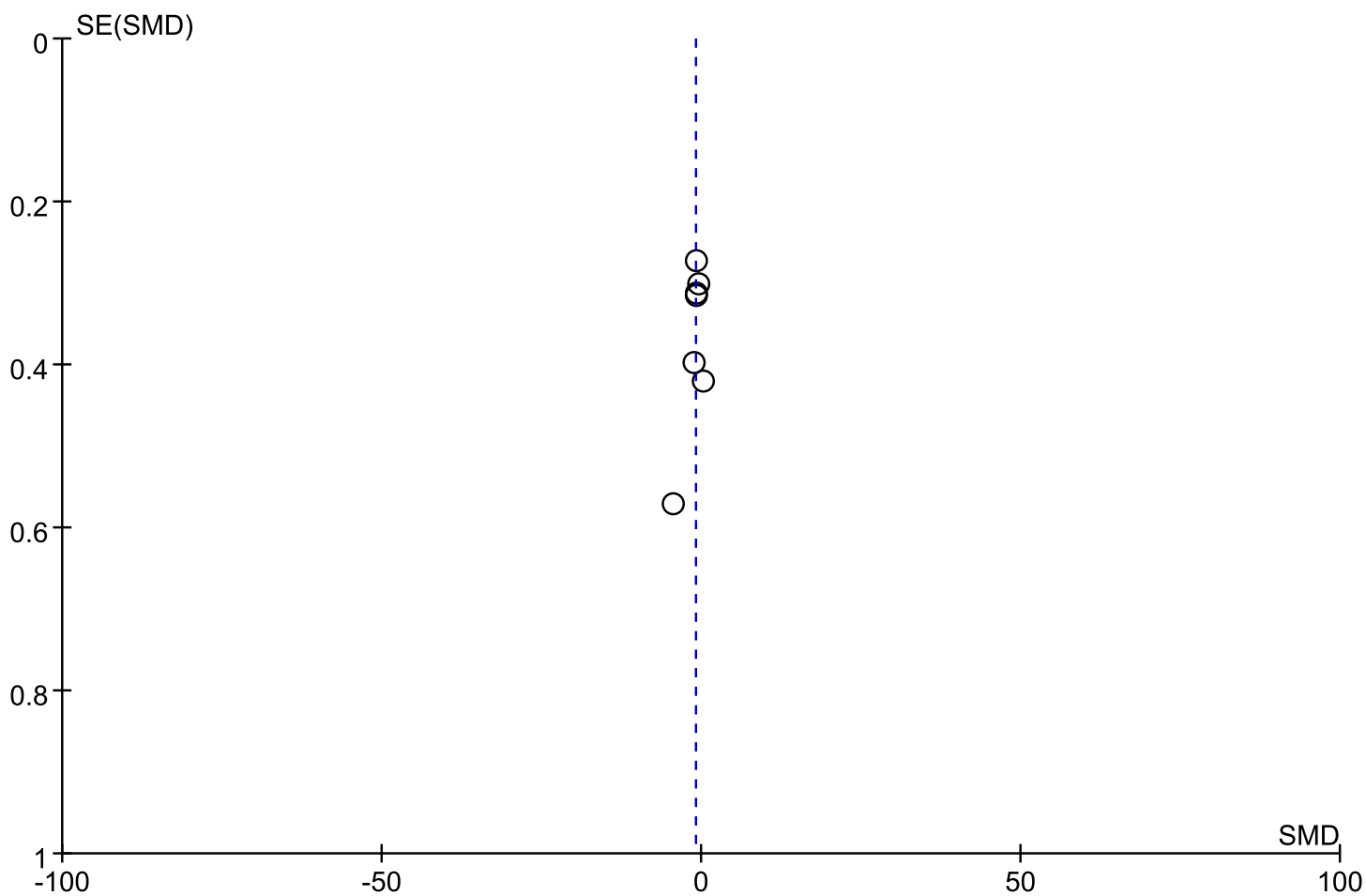

B

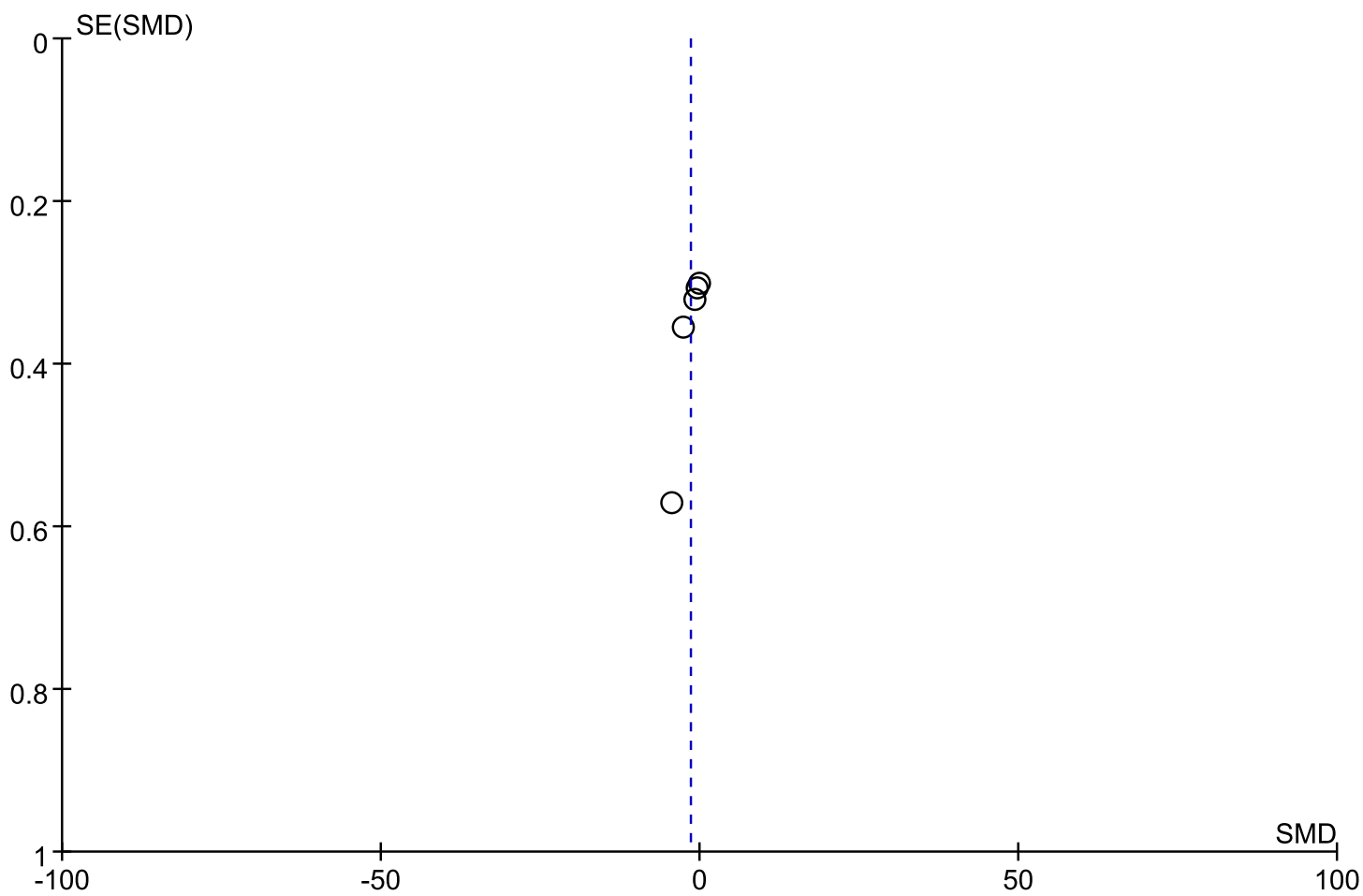

C

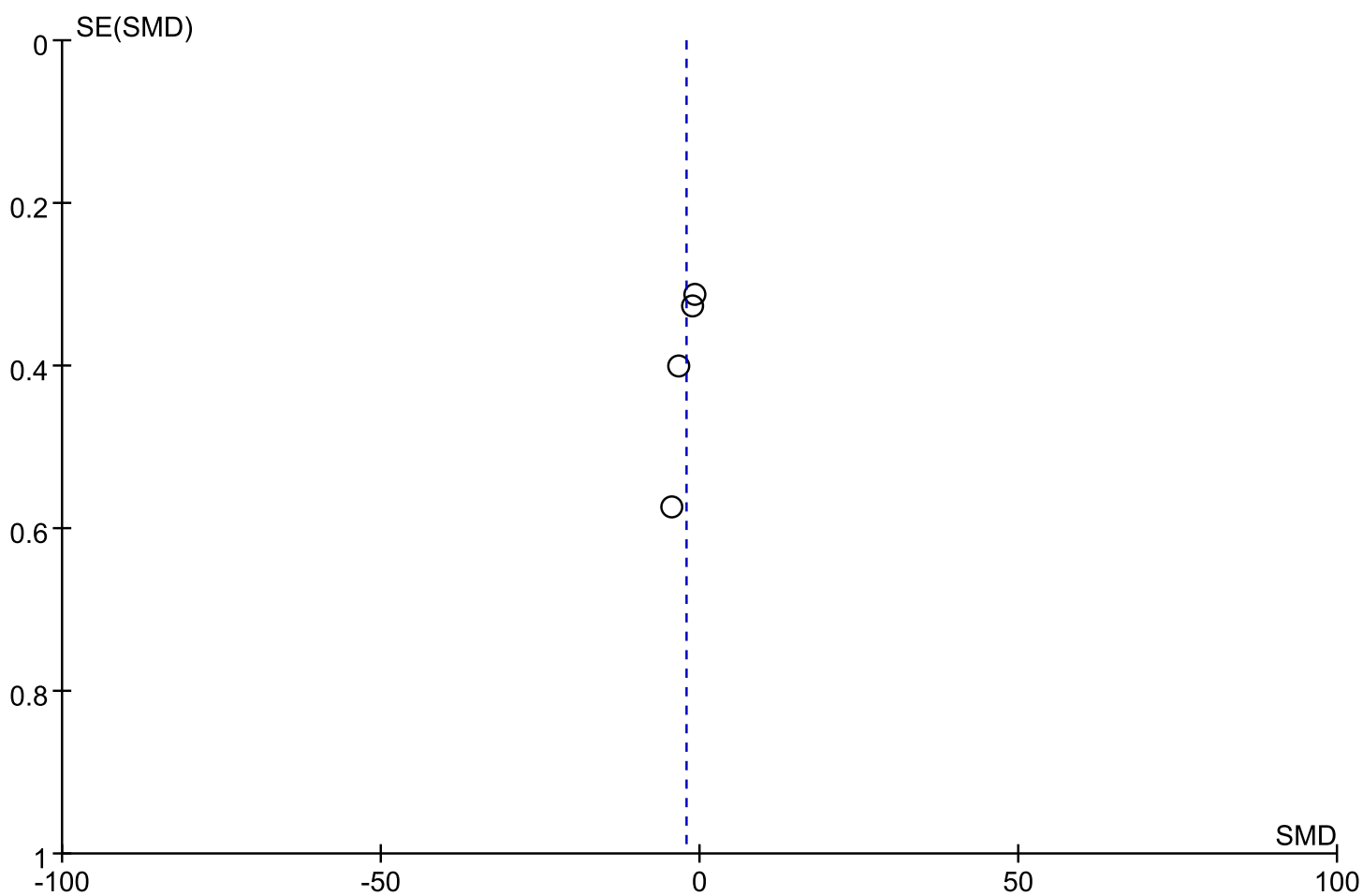

D

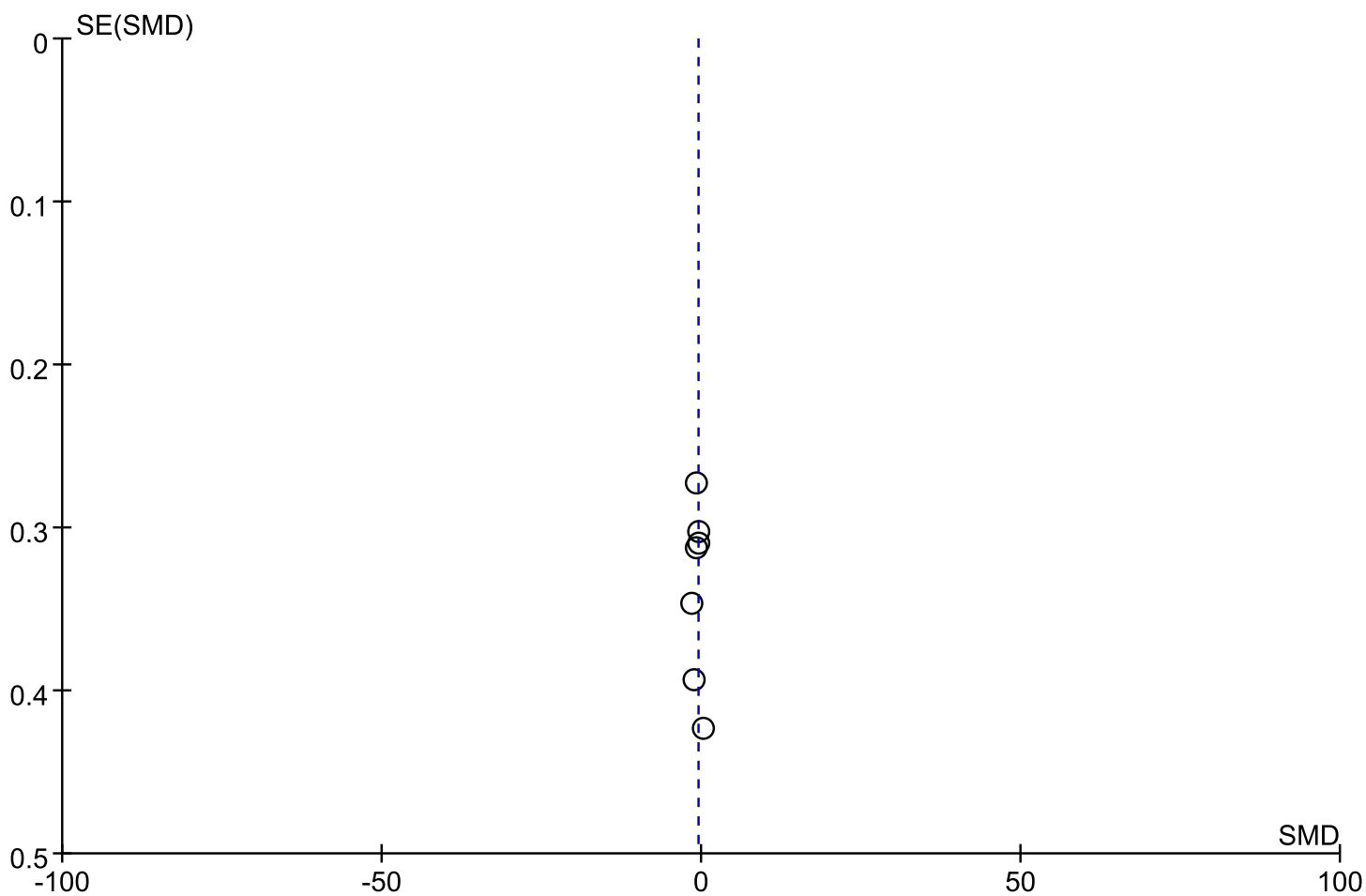

E

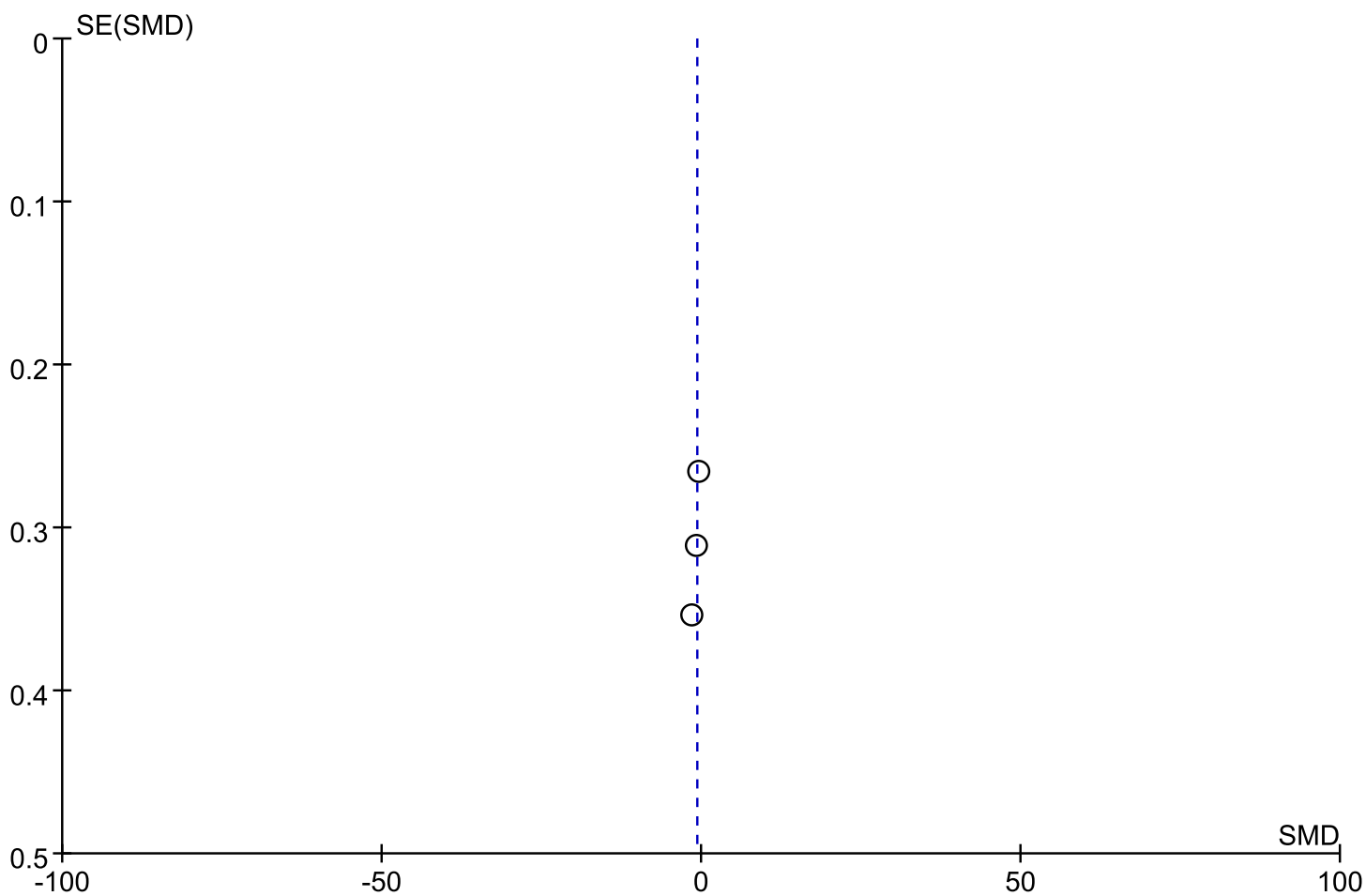

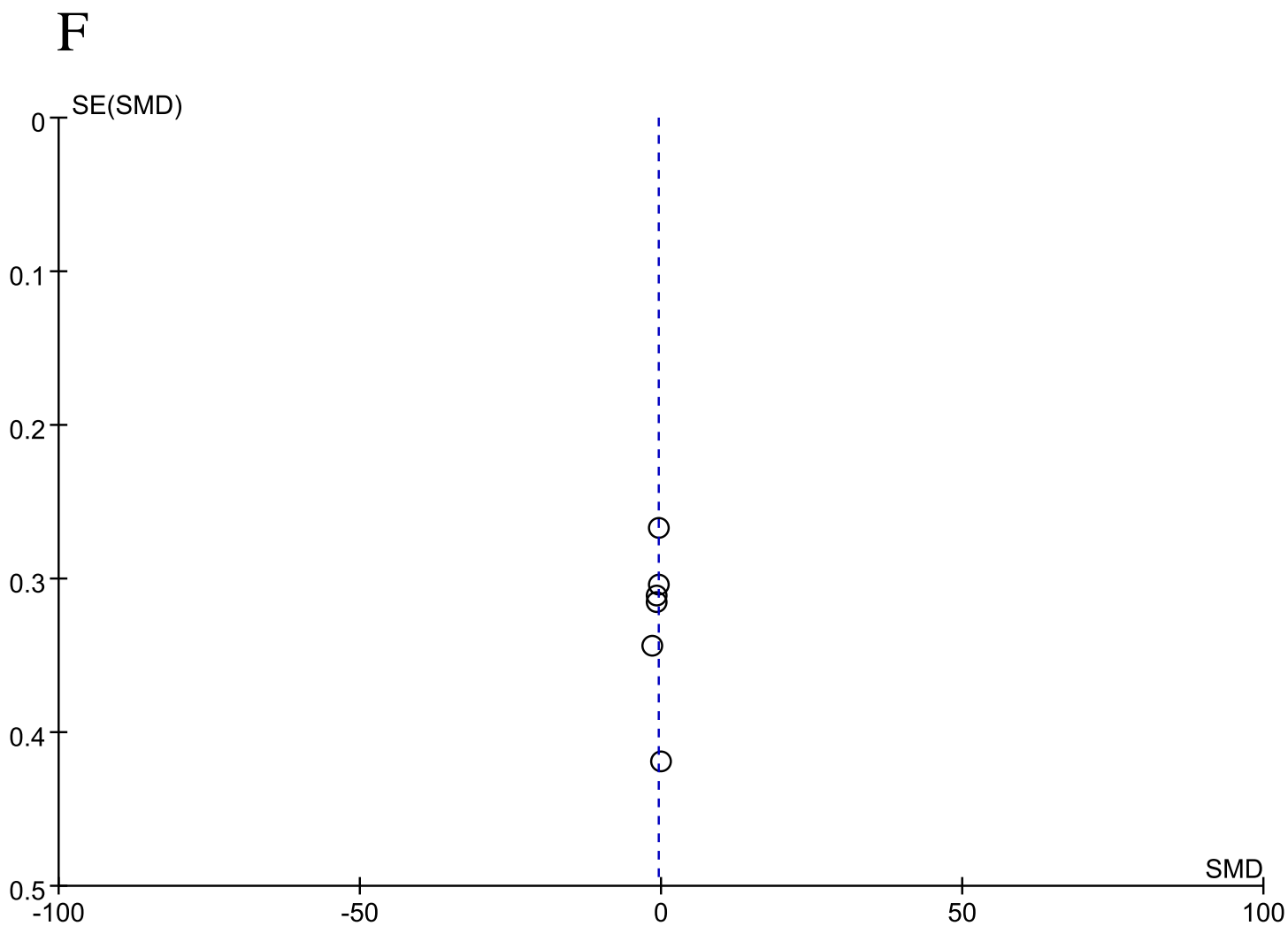

Supplement: Supplementary Materials — Supplementary Table S1: sensitivity analysis of meta-analysis. Supplementary Figure S1: meta-analysis of EA improving glucose and lipid metabolism. [file 5558665.f1.zip › Figure S1, Meta-analysis of EA improving glucose and lipid metabolism (1).pdf]
